# Effective and Efficient Porous CeO_2_ Adsorbent for Acid Orange 7 Adsorption

**DOI:** 10.3390/ma16072650

**Published:** 2023-03-27

**Authors:** Yaohui Xu, Liangjuan Gao, Jinyuan Yang, Qingxiu Yang, Wanxin Peng, Zhao Ding

**Affiliations:** 1 Laboratory for Functional Materials, School of New Energy Materials and Chemistry, Leshan Normal University, Leshan 614004, China; 2 Leshan West Silicon Materials Photovoltaic and New Energy Industry Technology Research Institute, Leshan 614000, China; 3 College of Materials Science and Engineering, Sichuan University, Chengdu 610065, China; 4 National Engineering Research Center for Magnesium Alloys, College of Materials Science and Engineering, Chongqing University, Chongqing 400044, China

**Keywords:** porous CeO_2_, template−free, adsorption, adsorption efficiency, acid orange 7

## Abstract

A porous CeO_2_ was synthesized following the addition of guanidine carbonate to a Ce^3+^ aqueous solution, the subsequent addition of hydrogen peroxide and a final hydrothermal treatment. The optimal experimental parameters for the synthesis of porous CeO_2_, including the amounts of guanidine carbonate and hydrogen peroxide and the hydrothermal conditions, were determined by taking the adsorption efficiency of acid orange 7 (AO7) dye as the evaluation. A template−free hydrothermal strategy could avoid the use of soft or hard templates and the subsequent tedious procedures of eliminating templates, which aligned with the goals of energy conservation and emission reduction. Moreover, both the guanidine carbonate and hydrogen peroxide used in this work were accessible and eco−friendly raw materials. The porous CeO_2_ possessed rapid adsorption capacities for AO7 dye. When the initial concentration of AO7 was less than 130 mg/L, removal efficiencies greater than 90.0% were obtained, achieving a maximum value of 97.5% at [AO7] = 100 mg/L and [CeO_2_] = 2.0 g/L in the first 10 min of contact. Moreover, the adsorption–desorption equilibrium between the porous CeO_2_ adsorbent and the AO7 molecule was basically established within the first 30 min. The saturated adsorption amount of AO7 dye was 90.3 mg/g based on a Langmuir linear fitting of the experimental data. Moreover, the porous CeO_2_ could be recycled using a NaOH aqueous solution, and the adsorption efficiency of AO7 dye still remained above 92.5% after five cycles. This study provided an alternative porous adsorbent for the purification of dye wastewater, and a template−free hydrothermal strategy was developed to enable the design of CeO_2_−based catalysts or catalyst carriers.

## 1. Introduction

The rise of the synthetic dye industry led to a revolution in chemical technology in the mid to late 19th century. Synthetic dyes developed rapidly, production varieties increased, output soared and they basically replaced natural dyes in the 20th century. To date, synthetic dyes have been widely applied to the fields of textiles, papermaking, plastics, leather, rubber, paints, cosmetics, food, etc. [1,2]. The world is so beautiful and colourful with almost 700,000 tons of synthetic dyes; however, 10–15% of these are discharged into wastewater, resulting in water pollution [3,4,5]. In particular, many synthetic dyes, such as azo dye and benzidine dye, are not only toxic to aquatic organisms, but also carcinogenic and mutagenic to humans [6]. Therefore, many techniques have been applied to remove these dyes from aqueous solutions, such as adsorption [7,8], ultrafiltration [9], photocatalytic degradation [10], electrochemical degradation [11], advanced oxidation processes [12], biological processes [13], etc. Among these numerous physical, chemical and biological techniques, the adsorption method using porous materials is favoured in the treatment of dye wastewater because of its insensitivity to toxicants, its simplicity and ease of handling and its low−cost [14,15]. Traditional porous adsorbents, including activated carbon [16], zeolite molecular sieve [17], porous alumina [18] and natural clays [19], are commonly used for wastewater treatment. However, these traditional porous materials have drawbacks, such as low selectivity and slow adsorption kinetics [20,21]. For these reasons, a novel class of adsorbent materials is still desirable.

At present, numerous adsorbents have been widely studied for the removal of various dyes due to their excellent performance as advanced materials, such as metal oxides (including NiO [22], ZnO [23], Fe_3_O_4_ [24], TiO_2_ [25]), NiZnAl layered double hydroxides [26], montmorillonite [27], chitosan [28] and hydrogel [29]. Among all the adsorbent materials, ceria (CeO_2_) is a significant and promising candidate because of its good environmental compatibility and thermal stability [30,31]. Recently, CeO_2_ has been employed in water pollution control and synthetized by different chemical and physicochemical strategies [32,33]. In addition, CeO_2_ has also been widely used in many other fields, such as oxygen storage capacitors [34], solid oxide fuel cells [35], ultraviolet blocking materials [36], catalysts [37], etc. Moreover, CeO_2_ particles have a positive surface charge at circumneutral pH [38,39,40], which makes them suitable as adsorbents to remove anionic dyes, such congo red (CR) [41], acid orange 7 (AO7) [42], reactive orange 16 (RO16), methyl orange (MO) and mordant blue 9 (MB9) [43]. In particular, these anionic dyes, including electron−rich groups (sulfonate group, SO_3_^–^), can coordinate with the empty 4*f* orbital of the Ce ion on the CeO_2_ surface [44]. This complexation between these dye molecules and CeO_2_ is more stable than adsorption by electrostatic action. Hence, it is imperative to design highly efficient CeO_2_ adsorbents to remove these dyes from aqueous solutions. Micro/nano−porous CeO_2_ is a promising candidate for dye removal because of its rich channel structure and high surface area. Generally, the preparation of porous CeO_2_ involves a selection of soft or hard sacrificial templates, as well as a design process of evaporation or casting to eliminate these templates [45,46,47]. These tedious procedures not only increase the cost of experiments, but also easily cause secondary pollution.

Herein, we report a template−free strategy for the synthesis of a porous CeO_2_ adsorbent through a wet chemical process at room temperature combined with a hydrothermal process, in which Ce(NO_3_)_3_∙6H_2_O (cerium source), guanidine carbonate (precipitating agent), hydrogen peroxide (H_2_O_2_, oxidizing agent) and H_2_O (inorganic solvent) were used only as starting reagents. Additionally, the as−obtained CeO_2_ was utilized to adsorb the AO7 azo dye, and the optimal experimental parameters for the synthesis of porous CeO_2_, including the amounts of guanidine carbonate and hydrogen peroxide and the hydrothermal conditions, were determined by taking the adsorption efficiency of AO7 dye in an aqueous solution as the evaluation. The experimental data from the adsorption of AO7 dye onto porous CeO_2_ were fitted according to the thermodynamic and kinetic models, and the porous CeO_2_ still exhibited good adsorption performance after five consecutive regeneration cycles.

## 2. Experimental Procedure

### 2.1. Materials

Ce(NO_3_)_3_∙6H_2_O (99.95%) was supplied by Aladdin Co. Ltd. (Shanghai, China). Hydrogen peroxide (H_2_O_2_, ≥30%) and ethanol were supplied by Chengdu Kelong Chemical Co., Ltd. (Chengdu, China). Guanidine carbonate and acid orange 7 (AO7, 97.0%) were supplied by Shanghai Maclin Biochemical Technology Co., Ltd. (Shanghai, China). Distilled water was used in all experiments.

### 2.2. Synthesis of Porous CeO_2_

A precursor of cerium was first synthesized using a chemical precipitation method, and then converted into CeO_2_ through the oxidation of H_2_O_2_ at room temperature. Typically, the desired amounts of guanidine carbonate (4~16 mmol) were added to the Ce^3+^ aqueous solution (20 mL, 0.2 mol/L) under continuous magnetic stirring, and a white precipitate (Ce_2_(CO_3_)_3_∙8H_2_O) was generated immediately. Subsequently, the desired amount of H_2_O_2_ (1~5 mL) was added to the above white suspension, and the white suspension promptly turned orange, then the suspension was stirred for 1 h and aged for 24 h.

The final CeO_2_ product was obtained following a hydrothermal process. Typically, the above suspension was decanted into a 50 mL Teflon−lined stainless steel autoclave, which was heated and maintained for 24 h at a set temperature (120~200 °C). Note that distilled water was used to make a total volume of about 25 mL. Finally, the resulting pale yellow precipitate (CeO_2_) was washed with distilled water and ethanol, then dried in air at 80 °C for 24 h.

### 2.3. Characterization

The phases of samples were examined using a DX−2700 X−ray diffraction (XRD, Dandong, China). The morphologies and microstructures of the CeO_2_ samples were examined using a JSM−7500F scanning electron microscopy (SEM, JEOL, Tokyo, Japan) and a JEM−2100F transmission electron microscopy (TEM, JEOL, Tokyo, Japan). Nitrogen adsorption–desorption isotherms of the CeO_2_ samples were measured on an ASAP2460 (Micromeritics, Norcross, GA, USA).

### 2.4. Adsorption of AO7 Dye

AO7, a typical azo dye, was selected as the model target to evaluate the adsorption capacity of the final porous CeO_2_ product. First, AO7 aqueous solutions with different concentrations of 100~180 mg/L were configured as simulated wastewater, then 0.2 g as−obtained CeO_2_ was dispersed into 100 mL AO7 solution with a desired concentration. The above mixture was stirred with a constant agitation speed of 200 rpm at room temperature, and the suspension was withdrawn at regular intervals. After the solid–liquid separation, the absorbance of the supernatant was measured at the absorption wavelength of 485 nm using an U−3900 ultraviolet−visible spectrophotometer (Uv−vis, Hitachi, Tokyo, Japan). The adsorption efficiency (*η*_t_,%) and the adsorption amount (*q*_t_, mg/g) were calculated using Equations (1) and (2), respectively. The experimental data from the adsorption of AO7 dye onto porous CeO_2_ were fitted according to the Langmuir (Equation (3)) [48] and Freundlich (Equation (4)) [49] isotherm models.

(1) $$\eta_{t}=\frac{C_{0}-C_{t}}{C_{0}}\times 100$$

(2) $$q_{t}=\frac{(C_{0}-C_{t})V}{m}$$

(3) $$\frac{C_{t}}{q}=\frac{1}{K_{L}q_{m}}+\frac{C_{t}}{q_{m}}$$

(4) $$\log q_{e}=\frac{1}{n}\log C_{e}+\log K_{F}$$

where *C*_0_ (mg/L) is the initial concentration of AO7 aqueous solution, *C*_t_ (mg/L) is the concentration of AO7 aqueous solution at a given time *t*, *m* (g) is the mass of porous CeO_2_ absorbent (0.2 g), *V* (L) is the volume of AO7 aqueous solution (100 mL), *K*_L_ and *K*_F_ are the Langmuir and Freundlich adsorption constants, respectively. Moreover, the saturated adsorption amount (*q*_m_, mg/g) of AO7 could be obtained according to Langmuir linear fitting.

In order to investigate the thermal properties of the adsorption process, the Gibbs free energy change (Δ*G*^0^, KJ/mol) and thermodynamic equilibrium constant (*K*_0_, L/g) were evaluated using Equation (5), while the entropy change (Δ*S*^0^, J/mol·K) and enthalpy change (Δ*H*^0^, KJ/mol) were obtained using the linear fitting of the Van’t Hoff equation (Equation (6)) [50]. Meanwhile, to explore the kinetics characteristics of the adsorption process, the experimental data were evaluated using the pseudo−first−order (Equation (7)) and pseudo−second−order (Equation (8)) models, respectively [51]. The equilibrium adsorption amount (*q*_e1,cal_ and *q*_e2,cal_, mg/g) and rate constant (*k*_1_, 1/h and *k*_2_, g/mg∙h) could be evaluated using the plots of log(*q*_e1,cal−_*q*_t_) vs. *t* and *t/q*_t_ vs. *t*.

(5) $$\Delta G^{0}=-RT\ln K_{0}\;\;\;\;(K_{0}=\frac{q_{e}}{c_{e}})$$

(6) $$\log K_{0}=-\frac{\Delta H^{0}}{2.303R}\times \frac{1}{T}+\frac{\Delta S^{0}}{2.303R}$$

(7) $$\log(q_{e1,\mathrm{cal}}-q_{t})=-\frac{k_{1}}{2.303}t+\log q_{e1,\mathrm{cal}}$$

(8) $$\frac{t}{q_{t}}=\frac{1}{q_{e2,\mathrm{cal}}}t+\frac{1}{k_{2}q_{e2,\mathrm{cal}}^{2}}$$

## 3. Results and Discussion

**Figure 1:**
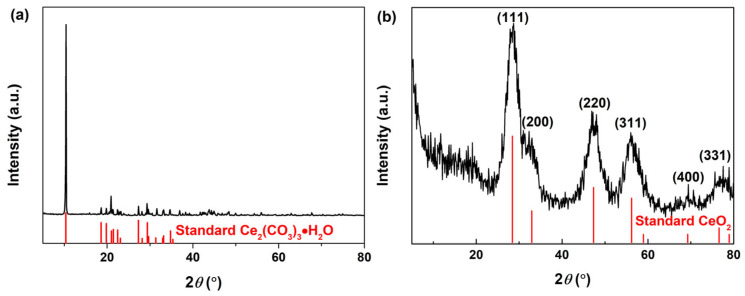
XRD patterns of the samples obtained following (**a**) addition of guanidine carbonate to the Ce^3+^ aqueous solution and (**b**) subsequent addition of 5 mL 30% H_2_O_2_.

Figure 1a shows the XRD pattern of the original white precipitate after adding guanidine carbonate to the Ce^3+^ aqueous solution. As observed, the obvious diffraction peaks in Figure 1a were assigned to the standard orthorhombic Ce_2_(CO_3_)_3_∙8H_2_O (JCPDS no. 38−0377), and this XRD pattern was similar to the commercial Ce_2_(CO_3_)_3_∙*x*H_2_O powders [52] obtained in previous studies [53,54]. After following the addition of 5 mL 30% H_2_O_2_, the XRD pattern in Figure 1b displayed several well−resolved peaks that could be indexed to (111), (200), (220), (311), (400) and (331) planes of the standard CeO_2_ with face−centred cubic structure (JCPDS no. 34−0394); however, its crystallinity was only 13.97% calculated using the X−ray diffraction method. Moreover, the diffraction peaks related to orthorhombic Ce_2_(CO_3_)_3_∙8H_2_O were no longer present, which indicated the complete transformation of orthorhombic Ce_2_(CO_3_)_3_∙8H_2_O into cubic CeO_2_ under the oxidation of H_2_O_2_.

**Figure 2:**
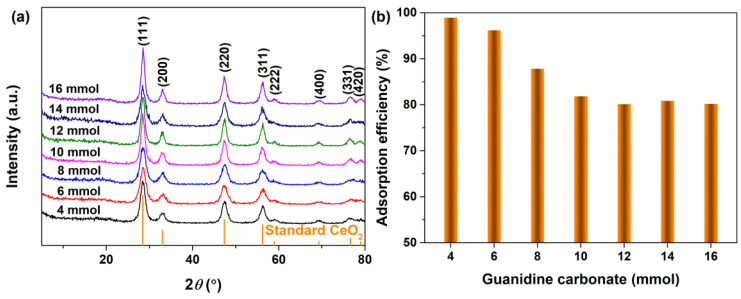
(**a**) XRD patterns of the hydrothermally synthesized CeO_2_ samples at 180 °C for 24 h with different addition amounts of guanidine carbonate (4~16 mmol) and 5 mL 30% H_2_O_2_. (**b**) Adsorption histograms of AO7 dye onto the as−obtained corresponding CeO_2_ in Figure 2a ([CeO_2_] = 2.0 g/L; [AO7] = 100 mg/L; *V* = 100 mL; 200 rpm; Room temperature; No pH preadjustment; *t* = 60 min).

Figure 2a shows the XRD pattern of the samples obtained with different amounts of guanidine carbonate (4~16 mmol) and 5 mL 30% H_2_O_2_ after hydrothermal treatment at 180 °C for 24 h. All patterns displayed several well−resolved peaks that could be indexed to (111), (200), (220), (311), (222), (400), (331) and (420) planes, which matched well with the standard CeO_2_ (JCPDS No. 34−0394) pattern. Moreover, the diffraction peaks of the CeO_2_ phase were complete and sharp, and no diffraction peaks of the impurity phase were observed, which suggested that pure CeO_2_ with a face−centred cubic structure was successfully synthesized through the synthesis strategy used in this work. Moreover, the optimal amount of ammonium carbonate was determined using the adsorption efficiency of CeO_2_ to AO7 dye in an aqueous solution under the same conditions. Figure 2b shows the corresponding adsorption histograms of AO7 dye onto CeO_2_ synthesized hydrothermally at 180 °C for 24 h with different amounts of guanidine carbonate (4~16 mmol) and 5 mL 30% H_2_O_2_. When the initial concentration of the AO7 aqueous solution was 100 mg/L, the adsorption efficiency achieved a maximum value of 98.92% for the CeO_2_ sample obtained with 4 mmol guanidine carbonate. With an increase in guanidine carbonate (6~12 mmol), the adsorption efficiency of AO7 by the as−obtained corresponding CeO_2_ decreased gradually, but was still higher than 80%. When the addition amount of guanidine carbonate was higher than 12 mmol, the adsorption efficiency remained basically unchanged. According to the above results, we concluded that the optimal addition amount of guanidine carbonate was 4 mmol for the synthesis of CeO_2_. Next, we investigated the influence of hydrothermal temperature on the phase composition of the samples and their adsorption efficiencies of AO7 dye.

**Figure 3:**
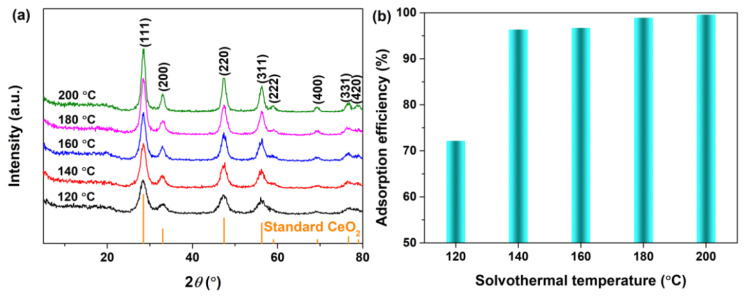
(**a**) XRD patterns of CeO_2_ synthesized at a set hydrothermal temperature of 120~200 °C for 24 h with 4 mmol guanidine carbonate and 5 mL 30% H_2_O_2_. (**b**) Adsorption histograms of AO7 dye onto the as−obtained corresponding CeO_2_ in Figure 3a ([CeO_2_] = 2.0 g/L; [AO7] = 100 mg/L; *V* = 100 mL; 200 rpm; Room temperature; No pH preadjustment; *t* = 60 min).

Figure 3a shows the XRD patterns of the CeO_2_ samples synthesized at a set hydrothermal temperature of 120~200 °C for 24 h with 4 mmol guanidine carbonate and 5 mL 30% H_2_O_2_. As observed in Figure 3a, all XRD patterns displayed several well−resolved peaks that could be indexed to the standard face−centred cubic CeO_2_ (JCPDS No. 34−0394), and no impurity phases were detected. With an increase in hydrothermal temperature, the corresponding diffraction peaks of as−obtained CeO_2_ sharpened gradually and their intensities also increased, which indicated that hydrothermal temperature could improve the crystallization of CeO_2_. Figure 3b shows the corresponding adsorption histograms of AO7 dye onto CeO_2_ synthesized hydrothermally at a set hydrothermal temperature of 120~200 °C for 24 h with 4 mmol guanidine carbonate and 5 mL 30% H_2_O_2_. When the initial concentration of the AO7 aqueous solution was 100 mg/L, the adsorption efficiency of the CeO_2_ synthesized at 120 °C was only 72.18%. With an increase in hydrothermal temperature, the adsorption efficiency of AO7 by CeO_2_ increased significantly, and achieved a maximum value of 99.59% for the CeO_2_ synthesized hydrothermally at 200 °C. Interestingly, the adsorption efficiencies of the CeO_2_ samples synthesized hydrothermally at temperatures above 140 °C were higher than 96%. Based on the above analyses, we concluded that the optimal hydrothermal synthesis temperature for CeO_2_ was 200 °C. We would next determine the optimal addition amount of H_2_O_2_ for the synthesis of CeO_2_.

**Figure 4:**
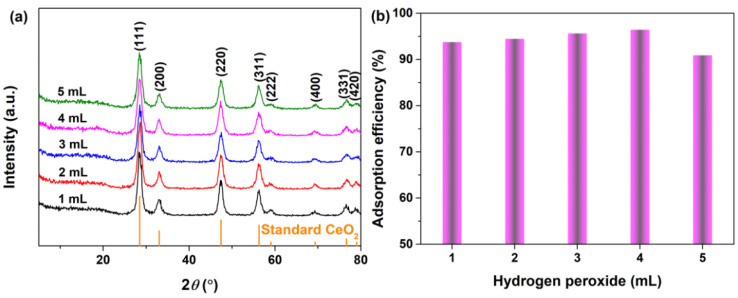
(**a**) XRD patterns of the hydrothermally synthesized CeO_2_ at 200 °C 24 h with 4 mmol guanidine carbonate and different additions of 30% H_2_O_2_ (1~5 mL). (**b**) Adsorption histograms of AO7 dye onto the as−obtained corresponding CeO_2_ in Figure 4a ([CeO_2_] = 2.0 g/L; [AO7] = 110 mg/L; *V* = 100 mL; 200 rpm; Room temperature; No pH preadjustment; *t* = 60 min).

Figure 4a shows the XRD patterns of CeO_2_ samples synthesized at 200 °C for 24 h with 4 mmol guanidine carbonate and different addition amounts of 30% H_2_O_2_ (1~5 mL). All the identified peaks in Figure 4a were assigned to the standard cubic CeO_2_ (JCPDS No. 34−0394), no impurity phases were detected and the intensities of the diffraction peaks of all the CeO_2_ samples were comparable. Figure 4b shows the corresponding adsorption histograms of AO7 dye onto CeO_2_ synthesized hydrothermally at 200 °C for 24 h with 4 mmol guanidine carbonate and different addition amounts of 30% H_2_O_2_ (1~5 mL). According to our previous adsorption experiment, the adsorption efficiencies of all the CeO_2_ samples for AO7 dye were close to 100% when the initial concentration of the AO7 aqueous solution was 100 mg/L, so we increased the initial concentration of AO7 solution to 110 mg/L. As observed in Figure 4b, the adsorption efficiency of CeO_2_ synthesized with 1 mL H_2_O_2_ was 93.75%. The as−obtained corresponding CeO_2_ synthesized with more H_2_O_2_ exhibited a slightly better adsorption of AO7, reaching a maximum value of 96.43% for the CeO_2_ synthesized with 4 mL H_2_O_2_. For the CeO_2_ synthesized with 5 mL H_2_O_2_, its adsorption efficiency decreased, but remained higher than 90%. Combined with the analysis results of XRD and the adsorption experiment in Figure 2, Figure 3 and Figure 4, the optimal experimental parameters for the synthesis of CeO_2_ were determined by taking the adsorption efficiency of AO7 as the evaluation: 4 mmol of guanidine carbonate, 4 mL of 30% H_2_O_2_ and a hydrothermal reaction at 200 °C for 24 h.

**Figure 5:**
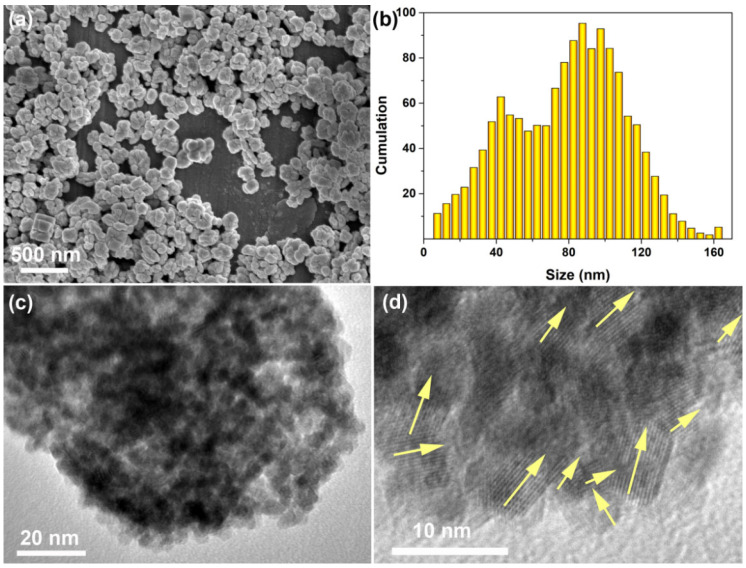
(**a**) SEM image, (**b**) size distribution histogram, (**c**) TEM and (**d**) HR−TEM images of CeO_2_ particles hydrothermally synthesized at 200 °C for 24 h with 4 mmol guanidine carbonate and 4 mL 30% H_2_O_2_. (The yellow arrows in (**d**) are the direction of lattice fringes).

The morphology of the CeO_2_ sample hydrothermally synthesized at 200 °C for 24 h with 4 mmol guanidine carbonate and 4 mL 30% H_2_O_2_ is shown in Figure 5a. As observed, the CeO_2_ featured equiaxed particles formed agglomerates. Moreover, the size value of the CeO_2_ particles was demonstrated using a statistical analysis, and the size distribution histogram is shown in Figure 5b. As observed, it was clearly found that most of the CeO_2_ particles were mainly concentrated at about 42.5 and 87.5 nm. Figure 5c shows the TEM image of a single CeO_2_ particle, which revealed the porous structure and the many pores around the nanoparticles. Moreover, the high−resolution transmission electron microscope (HR−TEM) image in Figure 5d shows that these nanoparticles had lattice fringes with the same direction (see the yellow arrows in Figure 5d), indicating the single crystal structure of these nanoparticles. 

**Figure 6:**
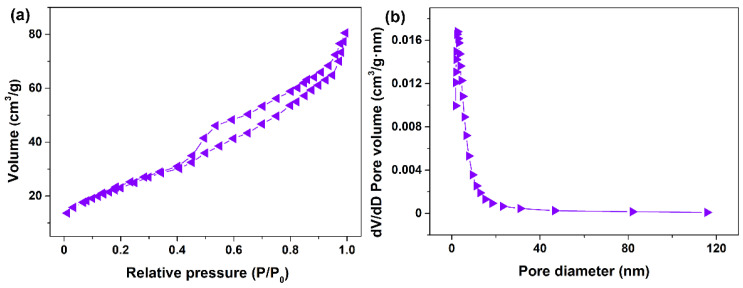
(**a**) N_2_ adsorption–desorption isotherm and (**b**) the corresponding Barrett–Joyner–Halenda pore size distribution curve of CeO_2_ hydrothermally synthesized at 200 °C for 24 h with 4 mmol guanidine carbonate and 4 mL 30% H_2_O_2_.

In order to further confirm the porous structure of CeO_2_, a N_2_ sorption experiment was performed, and the corresponding specific surface area, pore size and pore volume were determined. Figure 6a shows the N_2_ adsorption–desorption isotherm of the CeO_2_ hydrothermally synthesized at 200 °C for 24 h with 4 mmol guanidine carbonate and 4 mL 30% H_2_O_2_. Figure 6a shows that the N_2_ adsorption–desorption isotherm was similar to the Langmuir IV(a) type according to the IUPAC classification, and an obvious hysteresis loop was observed in the relative pressure (*P*/*P*_0_) range of 0.4~1.0, belonging to type H3 [55]. This isotherm was consistent with that of porous CeO_2_ in the reported literature [56,57,58], suggesting that the as−obtained CeO_2_ was a porous material with disordered mesoporous structures. The corresponding Barrett–Joyner–Halenda pore size distribution curve is shown in Figure 6b. The pore size presented a single distribution centred at about 2.5 nm, and the average pore size and pore volume were 6.2 nm and 0.129 cm^3^/g, respectively, using the Barrett–Joyner–Halenda analysis. Moreover, the specific surface area of mesoporous CeO_2_ was determined to be 86.8 m^2^/g using the Brunauer–Emmett–Teller method. 

**Figure 7:**
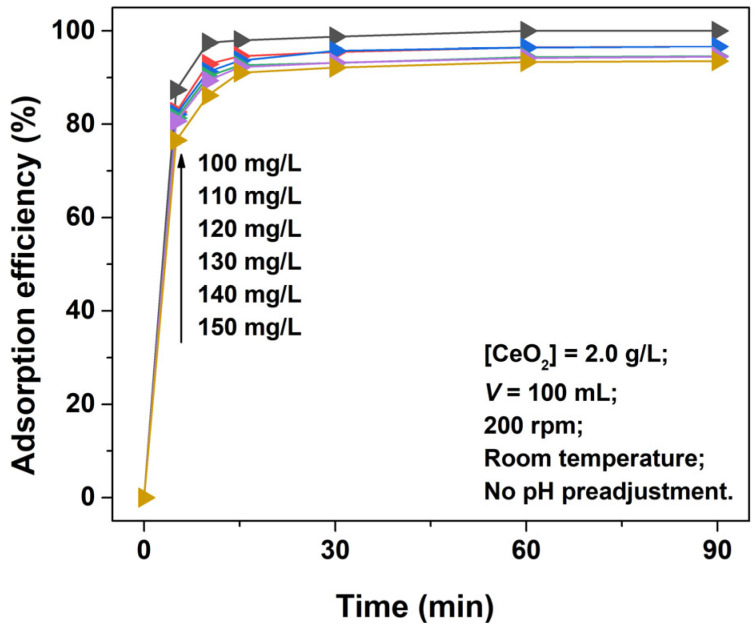
Time−dependence of the adsorption profiles of AO7 dye obtained at varying initial concentrations (100~150 mg/L) in the presence of porous CeO_2_ adsorbent hydrothermally synthesized at 200 °C for 24 h with 4 mmol guanidine carbonate and 4 mL 30% H_2_O_2_.

Figure 7 depicts the effects of the AO7 initial concentration (100~150 mg/L) on the adsorption efficiency of the porous CeO_2_ hydrothermally synthesized at 200 °C for 24 h with 4 mmol guanidine carbonate and 4 mL 30% H_2_O_2_. Figure 7 shows that the adsorption of AO7 was rapid for all the initial concentrations of the AO7 aqueous solution at the early stages of adsorption reaction. The adsorption efficiencies within 10 min of contact achieved 97.5, 92.9, 91.2, 90.2, 89.4 and 86.2% at AO7 initial concentrations of 100, 110, 120, 130, 140 and 150 mg/L, respectively. As the adsorption reaction continued, the adsorption process was mostly complete within 30 min. In other words, the adsorption–desorption equilibrium between porous CeO_2_ adsorbent and AO7 molecules was basically established within the first 30 min. The rapid and efficient adsorption of AO7 can be ascribed to the abundant porous structure of CeO_2_, which provides numerous adsorption sites for the AO7 molecule by increasing the effective contact area, and is helpful for transporting AO7 molecules to the adsorbent framework.

**Figure 8:**
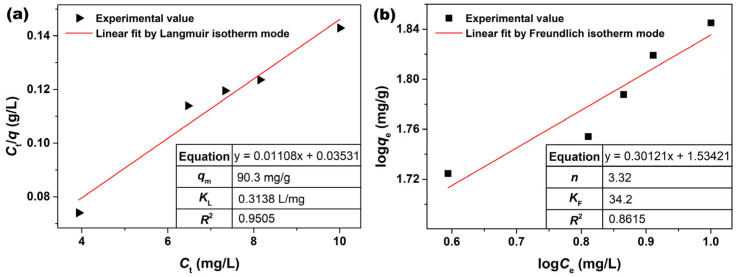
(**a**) Langmuir and (**b**) Freundlich linear fits of AO7 adsorbed onto porous CeO_2_ adsorbent hydrothermally synthesized at 200 °C for 24 h with 4 mmol guanidine carbonate and 4 mL 30% H_2_O_2_ ([CeO_2_] = 2.0 g/L; [AO7] = 110~150 mg/L; *V* = 100 mL; *t* = 60 min; 200 rpm; Room temperature; No pH preadjustment).

The experimental data from the adsorption of AO7 dye onto porous CeO_2_ were fitted according to the Langmuir and Freundlich isotherm models, and the linear fittings results are shown in Figure 8a,b, respectively. The corresponding Langmuir (*K*_L_) and Freundlich (*K*_F_) parameters calculated are listed in the insets in Figure 8a,b. The Langmuir isotherm model showed higher associated correlation coefficients (*R*^2^ = 0.9505) than that of the Freundlich isotherm model (*R*^2^ = 0.8615), which indicated that the Langmuir isotherm model was a better fit for modelling the AO7 adsorption onto porous CeO_2_. Moreover, the saturated adsorption amount (*q*_m_) of AO7 was 90.3 mg/g according to the Langmuir linear fitting. Furthermore, Table 1 shows the relevant literature on the development of adsorbents for AO7 removal. Among the existing adsorbent materials, activated carbons are the most commonly used and effective adsorbents for the removal of pollutants because of their abundant channels and high specific surface areas [59,60,61]. However, the preparation process of activated carbons has several disadvantages, including high energy consumption, high costs and can easily pollute the environment. For these reasons, endeavours have been made to develop alternatives to activated carbons, such as low−cost fly ash [62,63,64] and agro−residue [65,66]; however, their adsorption capacities are limited except for the brown coal fly ashes [67]. Other materials reported in the literature [16,22,68,69,70,71,72,73,74,75] exhibit satisfactory adsorption properties, especially 3D MgAl layered double hydroxide [75]. CeO_2_ and its complexes were also among the sequences being investigated. Compared to the reported CeO_2_ [42,76,77] and the porous CeO_2_ in our previous studies [53,54,78], the porous CeO_2_ in this work shows better adsorption capacity, but is lower than that of CeO_2_·*x*H_2_O [40]. It is worth noting that CeO_2_ with a porous structure not only has a potential application in the field of adsorption, but also in the fields of catalyst and catalysis carrier.

**Figure 9:**
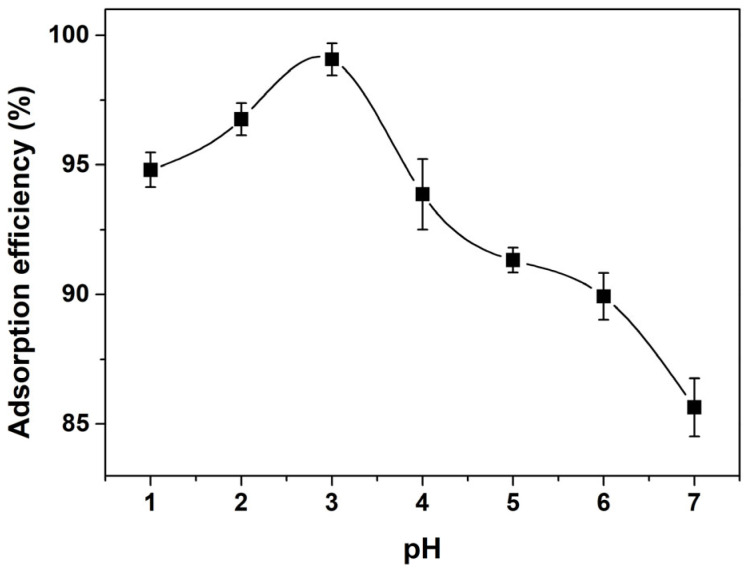
Effect of solution pH on the adsorption efficiency of AO7 onto porous CeO_2_ hydrothermally synthesized at 200 °C for 24 h with 4 mmol guanidine carbonate and 4 mL 30% H_2_O_2_ ([CeO_2_] = 2.0 g/L; [AO7] = 180 mg/L; *V* = 100 mL; *t* = 60 min; 200 rpm; Room temperature).

In order to determine the effect of the solution pH on the removal of AO7 dye onto porous CeO_2_ adsorbent, adsorption experiments, with varying pH levels of the AO7 aqueous solution in the range 1~7, were performed. As shown in Figure 9, with an increase in pH, the adsorption efficiency increased and reached its maximum when the pH value was about 3; the adsorption efficiency decreased with a continued increase in pH gradually. Moreover, a lower pH was conducive to the adsorption reaction. A possible reason for this could be that there were more available protons on the CeO_2_ surface at a lower pH, thereby increasing the electrostatic attraction between the negatively charged AO7 dye anions and positively charged CeO_2_, and causing an increase in adsorption. In contrast, the number of OH^−^ ions increased at higher pH values, which resulted in ionic repulsion between the negatively charged CeO_2_ surface and the anionic AO7 dye molecules. Considering the complexity associated with adjusting the pH of solution, as well as the possible environmental pollution risks, the subsequent adsorption experiments were carried out without pH preadjustment.

**Figure 10:**
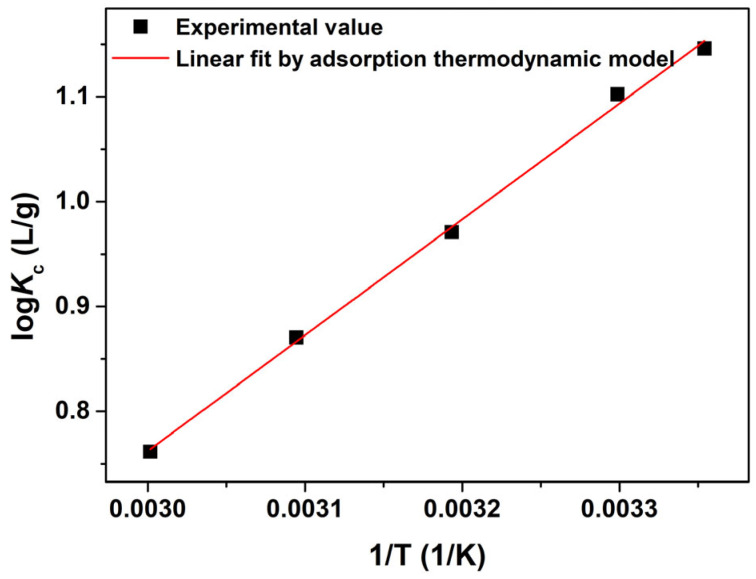
Experimental data from the adsorption of AO7 onto porous CeO_2_ fitted using the Van’t Hoff equation ([CeO_2_] = 2.0 g/L; [AO7] = 150 mg/L; *V* = 100 mL; *t* = 60 min; 200 rpm; Room temperature; No pH preadjustment).

The experimental data from adsorption at different temperatures were fitted using the Van’t Hoff equation, and the fitted linear curve is shown in Figure 10, while the thermodynamic parameters including *K*_0_, ∆*G*^0^, ∆*H*^0^ and ∆*S*^0^ are calculated and summarized in Table 2. Table 2 shows that *K*_0_ values decreased with an increase in temperature, which implies that the adsorption of AO7 molecules on the porous CeO_2_ surface was dominated by physical adsorption. The negative ∆*G*^0^ values at specified temperatures indicated that the adsorption reaction was spontaneous and favourable, while the negative ∆*H*^0^ value indicated that the adsorption reaction was exothermic. Furthermore, the negative ∆*S*^0^ value indicated that the three−dimensional motion of the AO7 molecules in solution transformed into two−dimensional motion on the CeO_2_ surface. Moreover, a high associated correlation coefficient (*R*^2^ = 0.9973) was obtained, confirming the reliability of the thermodynamic fitting result.

**Figure 11:**
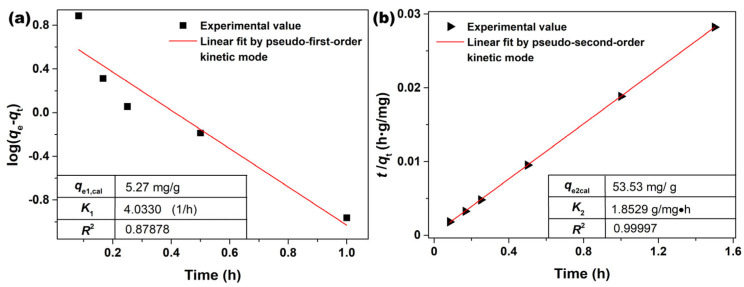
Fittings using (**a**) pseudo−first−order and (**b**) pseudo−second−order models for the adsorption of AO7 onto porous CeO_2_ ([CeO_2_] = 2.0 g/L; [AO7] = 110 mg/L; *V* = 100 mL; 200 rpm; Room temperature; No pH preadjustment).

The adsorption kinetics of AO7 molecules onto the porous CeO_2_ surface was tested using the pseudo−first−order and pseudo−second−order kinetic models; the linear fitting curves are shown in Figure 11. The kinetic parameters were calculated by plotting log(*q*_e_ − *q*_t_) vs. *t* (Figure 11a) and plotting t/*q*_t_ vs. *t* (Figure 11b), which are listed in Figure 11a,b as the insets. As observed in Figure 11, the pseudo−second−order model exhibited a better linear relationship than that of the pseudo−first−order, which was also supported by the higher correlation coefficients (*R*^2^ = 0.99997) of the pseudo−second−order model than that of the pseudo−first−order model (*R*^2^ = 0.87878). Combined with thermodynamic analysis, it can be concluded that the AO7 adsorption process involved not only physical adsorption, but also chemical adsorption.

**Figure 12:**
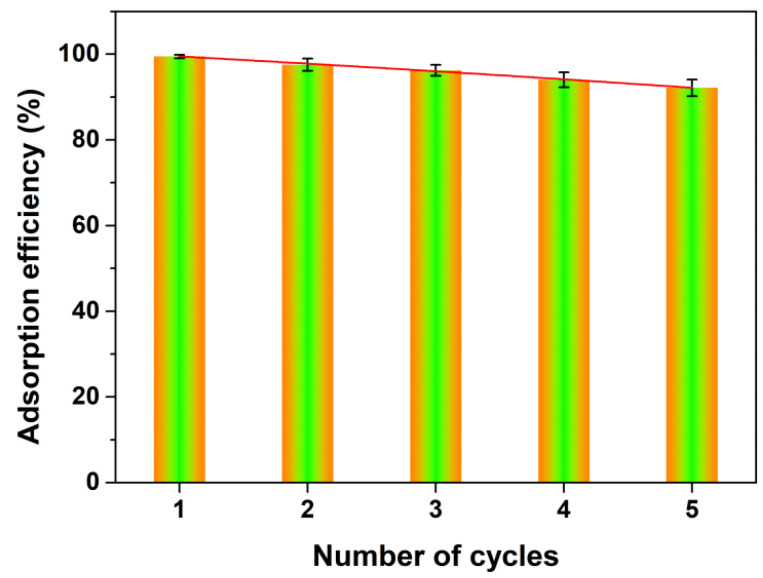
Adsorbent regeneration times on the adsorption efficiency of porous CeO_2_ ([CeO_2_] = 2.0 g/L; [AO7] = 100 mg/L; *V* = 100 mL; 200 rpm; Room temperature; No pH preadjustment).

To examine the reproducibility of the porous CeO_2_ absorbent in this work, five adsorption–desorption cycles were performed, in which a NaOH aqueous solution (0.6 mol/L, 20 mL) was employed as an eluant to desorpt AO7 molecules from the CeO_2_ surface. Figure 12 showed the adsorption histogram of five successive adsorption–desorption cycles. It was observed that the adsorption efficiency in the first adsorption–desorption cycle could reach 99.8%. The regenerated porous CeO_2_ adsorbent still exhibited a satisfactory uptake capacity, and the adsorption efficiency for AO7 remained at more than 92.5% after five cycles. The excellent adsorption properties and reproducibility of the porous CeO_2_ in this work suggested that they were suitable as a promising absorbent for dye removal in water.

## 4. Conclusions

A porous CeO_2_ adsorbent was successfully synthesized through a wet chemical process at room temperature, combined with a hydrothermal process in which Ce(NO_3_)_3_∙6H_2_O (cerium source), guanidine carbonate (precipitating agent), H_2_O_2_ (oxidizing agent) and H_2_O (inorganic solvent) were used only as starting reagents without an additional template. The optimal experimental parameters were determined by taking the adsorption efficiency of AO7 dye as the evaluation: 4 mmol of guanidine carbonate, 4 mL of 30% H_2_O_2_ and a hydrothermal process at 200 °C for 24 h. The porous CeO_2_ hydrothermally synthesized at 200 °C for 24 h, with 4 mmol guanidine carbonate and 4 mL 30% H_2_O_2,_ possessed an excellent adsorption capacity for AO7 dye. The adsorption–desorption equilibrium between CeO_2_ and AO7 molecules could basically be established within the first 30 min; in particular, the adsorption efficiencies within 10 min of contact could achieve 97.5% at an AO7 initial concentration of 100 mg/L. The saturated adsorption amount of AO7 dye was 90.3 mg/g according to fitting the experimental data with the Langmuir model. Moreover, while the CeO_2_ adsorbent could be recycled by using a NaOH aqueous solution, the removal percentage still reached 99.8% after the first cycle and remained above 92.5% after five consecutive adsorption–desorption cycles.

## Figures and Tables

**Table 1:** Recent literature on adsorbents for the removal of AO7 dye.

Adsorbent Name	Synthetic Method of Adsorbent	*q*_m_(mg/g) or Adsorption Rate (%)
Upflow packed−bed reactor containing activated carbon [60]	Activated carbon from Merck, granules of 2.5 mm	99% within 2 min (*C*_0_ = 110 mg/L)
Powdered activated carbon [61]	Procured from Merck	440 mg/g
Grade II fly ash [62]	Obtained from Huangpu Fuel Electric Plant, Guangzhou, China	1.10 mg/g
Fly ash [63]	Collected from coal fired boiler, and activated technique with heat treatment, alkali treatment and acid treatment.	3.14~12.72 mg/g
Bottom ash [64]	Procured from Bharat Heavy Electrical Limited in Bhopal, India.	68% (*C*_0_ = 35 mg/L)
Agro−residue (Soybean stalk) [65]	Grinding and screening	17.5 (pH = 2.0)
Agro−residue (Canola stalks) [66]	Grinding and screening	25.1 (pH = 2.5)
Brown coal fly ashes [67]	Collected at electrostatic precipitators in a power plant in the Czech Republic.	82.82 mg/g
Porous millimetre−sized amorphous TiO_2_/ZrO_2_ [68]	Template method and heating at 500 °C	>40
Multi−walled carbon nanotubes [69]	Floating catalyst chemical vapor deposition	47.72 mg/g
Iron oxide−loaded biochar [16]	Modification and pyrolysis at 600 °C	59.34 (pH = 6.0)
Activated carbon coated with ZnO [70]	Modification	66.22 mg/g
Zeolitic imidazolate framework−8 [71]	Wet chemical process at room temperature	80.47 mg/g (pH = 6.0)
Mesoporous activated carbon [72]	Heating milk vetch shrub at 600 °C	99.01 mg/g
One−dimensional mesoporous TiO_2_ nanotube [73]	Hydrothermal method and calcination at 400 °C	137.7 (pH = 3)
Magnetic mesoporous Fe−Ce bimetal oxides [74]	Hard template synthesis method	156.52 mg/g
Nickel (II) oxide [22]	Calcining nickel oxalate	178.57 (pH = 5.5)
3D MgAl layered double hydroxide [75]	Hydrothermal process	485.6 mg/g
CaO/CeO_2_ composite [42]	Co−precipitation process and annealing at 800 °C	92.68% (*C*_0_ = 10 mg/L)
CeO_2_ nanoparticles [76]	Hydrothermal procedure combined with calcination at 500 °C	~23% (*C*_0_ = 35 mg/L)
CeO_2_ powders [77]	Precipitation method combined with calcination at 500 °C	~56% (*C*_0_ = 35 mg/L)
Multilayered CeO_2_ microspheres [78]	Template−free solvothermal process combined with calcination at 500 °C	~99% (*C*_0_ = 35 mg/L)
Mesoporous CeO_2_ [53]	Template−free hydrothermal process	94.2% (*C*_0_ = 40 mg/L)
Mesoporous CeO_2_ [54]	Template−free hydrothermal process	90.07% (*C*_0_ = 100 mg/L)
CeO_2_·*x*H_2_O [40]	Precipitation method using NH_3_·H_2_O as a precipitant	164 mg/g
Porous CeO_2_ in this work	Template−free hydrothermal process	~100% (*C*_0_ = 100 mg/L)

**Table 2:** Thermodynamic parameters for the adsorption of AO7 onto porous CeO_2_ hydrothermally synthesized at 200 °C for 24 h with 4 mmol guanidine carbonate and 4 mL 30% H_2_O_2_ ([CeO_2_] = 2.0 g/L; [AO7] = 150 mg/L; *V* = 100 mL; *t* = 60 min; 200 rpm; Room temperature; No pH preadjustment).

Δ*G*^0^ (KJ/mol)					*K*_0_ (L/g)					Δ*H*^0^ (KJ/mol)	Δ*S*^0^ (J/mol·K)	*R* ^2^
25 °C	30 °C	40 °C	50 °C	60 °C	25 °C	30 °C	40 °C	50 °C	60 °C			
−6.54	−6.40	−5.82	−5.38	−4.86	14.00	12.66	9.35	7.42	5.78	−21.15	−48.87	0.9973

## Data Availability

Not applicable.
